# Shellfish toxicity in UK waters: a threat to human health?

**DOI:** 10.1186/1476-069X-8-S1-S12

**Published:** 2009-12-21

**Authors:** Keith Davidson, Eileen Bresnan

**Affiliations:** 1 Scottish Association for Marine Science, Dunstaffnage Marine Laboratory, Oban Argyll, PA37 1QA, UK; 2 Marine Scotland – Science, 375 Victoria Road, Aberdeen, AB11 9DB, UK

## Abstract

The potential for poisoning of humans through their consumption of shellfish which have themselves consumed biotoxin producing marine phytoplankton exists in the UK. Toxins are bio-accumulated within the shellfish flesh allowing them to reach harmful concentrations. This threat is in most part mitigated by monitoring programmes that assess both the presence of potentially harmful phytoplankton and shellfish flesh toxicity. However, the medical profession in the UK remains relatively ignorant of the potential for biotoxin derived shellfish toxicity, preventing quantification of magnitude, frequency, and severity of health effects in the community or the medical significance of more recently discovered toxins. While the current causative species and their toxins are relatively well characterised there remains a lack of understanding of the factors governing the temporal and spatial appearance of harmful phytoplankton. Expansion of shellfish aquaculture is likely both worldwide and in the UK. Better understanding of how harmful phytoplankton interact with their environment to promote the sporadic harmful blooms that we observe is required to underpin risk assessments.

## Introduction

A variety of phytoplankton species in UK waters are responsible for the production of biotoxins. Filter feeding shellfish accumulate these toxins within their flesh, posing a risk to human health if they are consumed. Shellfish harvesting areas in UK waters are subject to closure due to the detection of high concentrations of toxins responsible for three shellfish poisoning syndromes: paralytic shellfish poisoning (PSP), diarrhetic shellfish poisoning (DSP) and amnesic shellfish poisoning (ASP). Toxins associated with lipophilic shellfish toxins (LSTs) and spirolides have also been detected but pose less of a problem. In this short paper, based on the proceedings of a science/industry workshop "Relating Harmful Phytoplankton to Shellfish Poisoning and Human Health" held in October 2007, we summarise the status of shellfish toxins and human health in UK waters. Further information and references can be found in the full workshop report [1].

## Causative organisms & toxins

**Figure 1:**
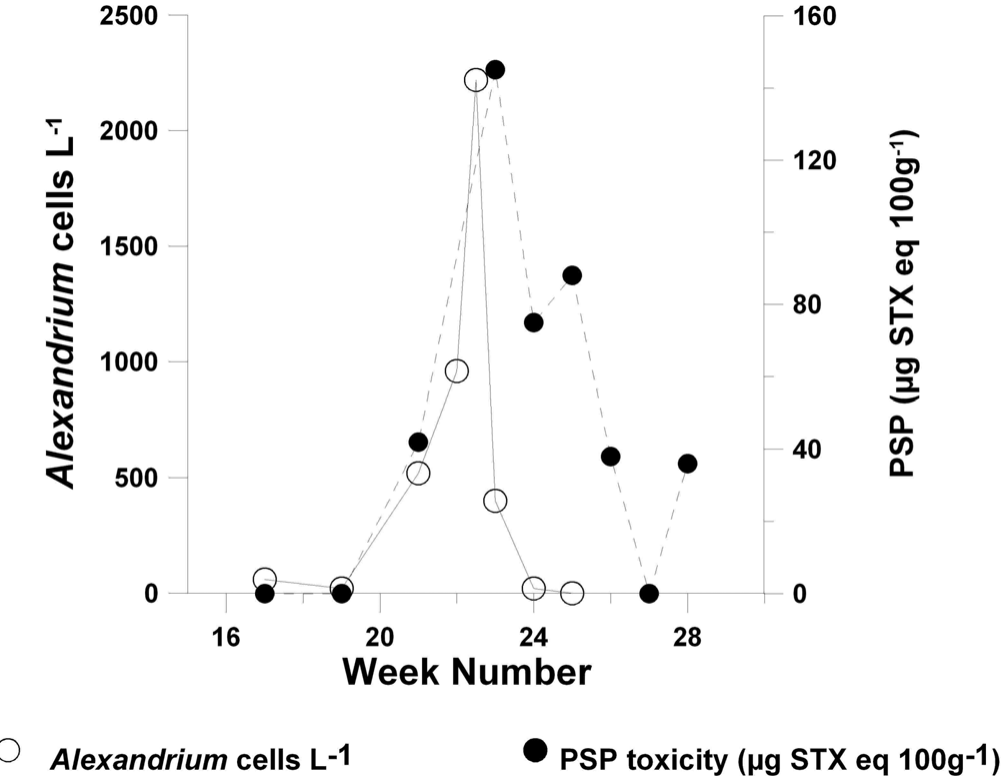
***Alexandrium *cell numbers and PSP toxicity in the blue mussel (*Mytilus edulis*) from the Orkney Islands during 1997**.

PSP toxins are produced by the dinoflagellate genus *Alexandrium *with *A. tamarense *(Group I) identified as a potent PSP producer in Scottish waters [2,3] and *A. minutum *has been identified as a PSP producer in England [4]. *A. ostenfeldii *from both Scottish and English waters has been observed to produce trace amounts of PSP as well as spirolides [4,5]. PSP intoxication of shellfish is regional in nature with particular hotspots including the Orkney and Shetland Islands. In these regions relatively low cell densities of *Alexandrium *(< 2,000 cells L^-1^) have been associated with closures of blue mussel (*Mytilus edulis*) harvesting areas following shellfish toxicity above the regulatory limit (Figure 1). In contrast, PSP does not cause major problems in other areas of the UK.

**Figure 2:**
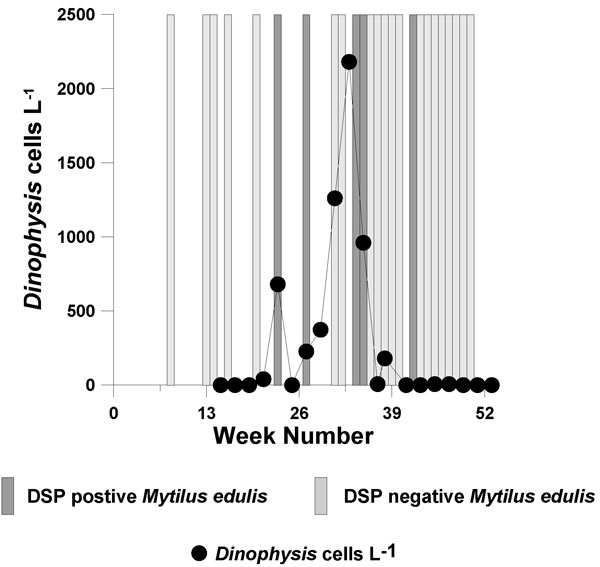
***Dinophysis *cells numbers and DSP results (positive/negative bioassays) in the blue mussel (*Mytilus edulis*) from a sea loch on the west coast, Scotland during 2001**.

DSP toxins are associated with the dinoflagellate genus *Dinophysis*. The main species observed are *D. acuminata *and *D. acuta*. Considerable inter-annual variation has been observed with *D. acuta *dominating in Scottish waters in 2001. Since then, numbers have subsequently declined with *D. acuminata *becoming more dominant. DSP toxins are recorded on an annual basis in Scotland (Figure 2) but tend to be less frequently detected in other UK regions. The dinoflagellate *Prorocentrum lima*, also associated with DSP, has been detected at selected sites around the UK [6,7]. Due to its epiphytic nature, this species may be under represented in sampling programmes.

ASP is associated with diatoms of the genus *Pseudo-nitzschia*. Thirteen *Pseudo-nitzschia *species are present in UK waters [8,9] but only *P. australis *and *P. seriata *are confirmed as toxin producers in Scottish waters [10] along with *P. multiseries *from English waters [4]. ASP toxins accumulate in King Scallops resulting in extensive closures [11]. However toxicity of wild scallops has effectively been mitigated by separation of the shellfish tissue and end product testing which allows non toxic parts of the scallop flesh to be marketed. Monitoring of other shellfish continues in the UK however closures due to high concentrations of ASP are rare in these shellfish.

LSTs are produced by a range of dinoflagellates. *Dinophysis *spp. are linked to pectenotoxins (PTX), while *Lingulodinium polyedrum, Protoceratium reticulatum *and *Gonyaulax grindleyii *implicated in yessotoxin (YTX) production. Recently a small dinoflagellate, Azadinium spinosum, has been implicated in azaspiracid (AZA) production [12]. Analysis of shellfish using liquid chromatography-mass spectrometry (LC-MS) methodology demonstrated the presence of multiple LSTs at low concentrations within Scottish shellfish [13].

A final species of interest is *Prorocentrum minimum*, which can occasionally form dense blooms. The toxicity of this species in UK waters has yet to be investigated.

## Health risk and causative factors

Both PSP and ASP are potent neurotoxins, both of which have been responsible for human fatalities in other countries [14]. PSP is a sodium channel blocker that can cause paralysis which, in severe form, can lead to death. ASP destroys the brain cells that release the neurotransmitter glutamic acid and can have a severe impact on the elderly [14]. DSP, while less dangerous, is characterised by severe gastrointestinal symptoms which can impact on the frail or elderly. Prevention of shellfish poisoning is therefore important for public health and also to ensure the sustainable development of the shellfish industry which is often of considerable importance to the economy of rural areas.

Shellfish toxin contamination is usually acute, sporadic and difficult to predict, as the factors governing the proliferation of harmful phytoplankton are poorly understood. However, at least in UK waters, harmful blooms are most probably part of the variability of marine flora with the evidence for anthropogenic nutrient enrichment as the causative factor being limited. Rather, hydrographic, meteorological, and chemical factors interact to promote blooms [15].

## Monitoring

Monitoring of shellfish flesh for the presence of toxins began in response to a PSP event in the North East of England in 1968 [16]. However, during the 1990s, with the implementation of the EU Shellfish Hygiene Directive, monitoring expanded geographically also including phytoplankton cell counts. Phytoplankton analysis is performed weekly using the Utermöhl technique [9,16] while toxicity analysis is performed on a weekly or monthly basis on a risk assessed basis [17]. While toxicity is still detected in shellfish, regulatory monitoring has been generally successful in preventing contaminated product reaching the marketplace.

## Discussion

Worldwide, consumption of marine products continues to expand; having now surpassed other animal protein sources [18]. Landings of bivalves in the UK are estimated by the Shellfish Association to be ~€67 million in 2006, much of which is exported [19]. Aquaculture is rapidly growing in importance, now contributing ~25% of fish/shellfish for consumption. With the global decline in wild fish stocks, it is inevitable that increased aquaculture production will continue, thus it is critical to ensure the safety of the product without undermining public confidence in it.

Monitoring in UK waters has been generally successful in safeguarding humans from shellfish poisoning. However, while the threat posed by cyanobacterial toxins from inland waters has been recognised by medical practitioners, the risk associated with marine biotoxins is less well appreciated. While this may have led to under-reporting and recording of shellfish poisoning events, it may equally have contributed to an unjustified but common public perception of shellfish consumption being "risky". Quantitative evidence of human intoxication levels and understanding of the magnitude of any health risk set against the health benefits of shellfish consumption is therefore a challenging but important research priority. Furthermore, while increasing ability to detect and quantify toxins is reassuring, it is important that research to quantify the concentrations of these toxins at which they are medically significant to humans keeps pace, preventing "scaremongering" or unnecessary harvesting closures.

As harmful blooms typically develop rapidly, risk assessment methodologies are required to allow the industry to better plan harvesting operations at times of lower risk. While it is clear that some general patterns exist, harmful phytoplankton exhibit spatial and temporal variability. Such heterogeneity suggests that local risk assessments based on detailed knowledge of the physiology of the causative species and hydrography and meteorology of the local environment are most likely to be successful.

Finally, there remains a need to be vigilant for invasive species. Species such as the PSP producer *Gymnodinium catenatum*, that affect the Galician coast, are currently not detected in UK waters. Given the increase in sea surface temperatures in the region as well as the potential for the introduction of new species via ship's ballast, it will be important for monitoring agencies to familiarise themselves with the identification of potential invasive species.

## Note

The peer review of this article can be found in Additional file 1.

## Competing interests

The authors declare that they have no competing interests.

## Authors' contributions

KD and EB coordinated the first UK HAB Workshop, Oban, 15th - 16th October 2007. Both authors summarised the historical data available and output from this workshop to produce this review. The contribution of all workshop participants are acknowledged.

## Supplementary Material

Additional file 1 Peer review. Click here for file
