# Supplementary material for: Shellfish toxicity in UK waters: a threat to human health?
Source: Environ Health. 2009 Dec 21;8(Suppl 1):S12. doi: 10.1186/1476-069X-8-S1-S12 (PMC2796490; doi:10.1186/1476-069X-8-S1-S12)
Supplement: Additional file 1 — Peer review. [file 1476-069X-8-S1-S12-S1.doc]

| Article ref no. | **A1471-2105X SZ-S12** |
| --- | --- |
| Title | **Shellfish toxicity in UK waters: a threat to human health** |
| Author(s) | Keith Davidson & Eileen Bresnan |

**Ref#1**

**General comments:** This is a very useful short review of shellfish toxicity in UK. It contains pertinent information on causative phytoplankton species which would have been available previously only in reports.

Some sections appear overly brief with insufficient detail: Health risk; Monitoring. But I do not know if there is a short page limit for this issue.

My other concern is that there may be a Scottish bias in this review and references, though I understand that this may appropriate given the more frequent and harmful blooms there.

**Major compulsory revisions:**

None.

**Minor essential revisions:**

P1 Abstract: Change ‘medial’ to ‘medical’.

P2 Intro: “Toxins,” remove comma.

P3 para 3: Explain term “shucking”.

P6 para 2: “Un-necessary” remove hyphn.

Figures 1 and 2 legends: both missing year of data.

P4 Health risk: provide additional detail, particularly explaining the causative factors.

P5 Monitoring: provide additional detail, particularly explaining methods and frequency of toxin and phytoplankton monitoring.

**Discretionary revisions:**

This paper would benefit from additional figures, in particular a UK map showing the relative distribution of HAB events / algal toxins / HAB phytoplankton species.

**Ref#2**

**General comments:** Good short overview of algal toxicity in the UK.

**Minor essential revisions:**

Abstract: Change medial to medical

# Response to Referees’ comments

*General comments:*

Sections on Heath Risk and Monitoring have been reworded to provide required detail. Shellfish toxicity is more prevalent in Scottish waters with more information readily available in the literature. This is why these examples and references have been used.

*Reviewer 1:*

All minor essential revisions completed. On P4 and P5 where more information was requested, relevant sections were reworded with references included to provide the additional detail. Additional figures (suggested under the discretionary revisions) were not included owing to the two figure limit per manuscript.

*Reviewer 2:*

All minor essential revisions completed.
